# Liquid Metal Machine Triggered Violin‐Like Wire Oscillator

**DOI:** 10.1002/advs.201600212

**Published:** 2016-08-17

**Authors:** Bin Yuan, Lei Wang, Xiaohu Yang, Yujie Ding, Sicong Tan, Liting Yi, Zhizhu He, Jing Liu

**Affiliations:** ^1^ Key Laboratory of CryogenicsTechnical Institute of Physics and ChemistryChinese Academy of SciencesBeijing100190P. R. China; ^2^ Department of Biomedical EngineeringSchool of MedicineTsinghua UniversityBeijing100084China

**Keywords:** hybrid structures, liquid metal machines, self‐actuation, wetting, wire oscillators

## Abstract

**The first ever oscillation phenomenon of a copper wire embraced inside a self‐powered liquid metal machine** is discovered. When contacting a copper wire to liquid metal machine, it would be swallowed inside and then reciprocally moves back and forth, just like a violin bow. Such oscillation could be easily regulated by touching a steel needle on the liquid metal surface.

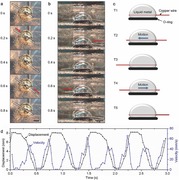

Oscillation is a widely seen dynamic phenomenon^1^, ^2^, ^3^ in mechanical,^4^ electrical,^5^ biological,^6^ and chemical^7^ systems. Oscillatory chemical reactions such as Belousov–Zhabotinsky reaction^8^ and mercury beating heart^9^ are classical examples of nonequilibrium thermodynamics switching between different patterns. Here we found the first ever oscillation phenomenon of a copper wire embraced inside the liquid metal machine via chemical and mechanical coupling. Recently, it was revealed that gallium‐based liquid metal owns a rather important value to serve as shape transformable material^10^, ^11^ due to its unique property of high electrical conductivity, excellent fluidity, and low melting point. In addition, feeding the liquid metal with aluminum would lead to self‐powered motors which could keep long‐term actuation performance in alkaline solution^10^ due to surface tension gradient and H_2_ propulsion mechanism. The present study discovered even more unusual effects that apart from self‐actuation, such a liquid metal machine would trigger a copper wire to reciprocally move back and forth across the liquid metal body. When contacting a copper wire to the liquid metal motor, it will be wetted and swallowed and then oscillates horizontally like a violin bow at the frequency of about 1.2 Hz. Moreover, the oscillation could be easily regulated and speeded up by touching a steel needle on the liquid metal motor surface. This fundamental phenomenon can be explained by the wetting behavior difference due to chemical reaction. Given appropriate designing, such an autonomous oscillator composed of hybrid solid and liquid metal structures can be developed as a core switch element in periodically regulating devices to realize various particular fluidic, electrical, mechanical, and optical functions. The present finding refreshes the basic understanding of the soft machines commonly conceived in textbook as well as adds new knowledge to the wetting science.^12^ It also opens a basic way to fabricate a self‐powered wire oscillator using liquid metal as the main machine body.

The liquid metal containing aluminum granules was placed in a rubber O‐ring (diameter 10.6 mm) to avoid self‐motion.^10^ A copper wire (length 20 mm and diameter 0.19 mm) inserted inside the liquid metal oscillated horizontally from side to side (**Figure** **1** and Supporting Information Videos S1 and S2). The oscillation motion could last for about half an hour. The whole system was immersed in aqueous NaOH solution (0.5 m). Hydrogen was mainly generated from the bottom of the liquid metal^13^ and the copper wire surface contacting the solution (Figure 1a,b). The H_2_ generation on copper wire surface was mainly from Al granules deposited on wire surface when it went through liquid metal. It can be proved by the fact that when the copper wire was pulled out of the liquid metal, the surface still kept producing H_2_ for about 2 min (Extended Data Figure S1 and Video S3, Supporting Information). Particularly, the middle part of the wire produced H_2_ for a longer time since it was embedded inside the liquid metal all the time with almost no Al consumption. This process resembled the monolayer deposition as found before.^14^ Apart from the copper wire, we also carried out a group of comparative experiments to test whether more metal wires can work as the oscillator candidates. Particularly, Ni, Ti, Ag, and steel wires were investigated. However, as indicated by the experiments, the liquid metal could not wet Ni, Ti, and steel wires while Al granules would not attach on the Ag wire surface. Therefore, all these tested three wires were not suitable for making an oscillating machine, although more other materials can be tried in the near future. For brief and also for such a reason, only a copper wire machine will be focused in the present study. According to the experiments, after the copper wire moved to one edge of the liquid metal machine, it would stay there for a while before moving back to the other edge. The period of the oscillation was about 0.8 s. The phase portrait of the oscillation is shown in Extended Data Figure S2 (Supporting Information), which resembled the harmonic oscillation.

**Figure 1:**
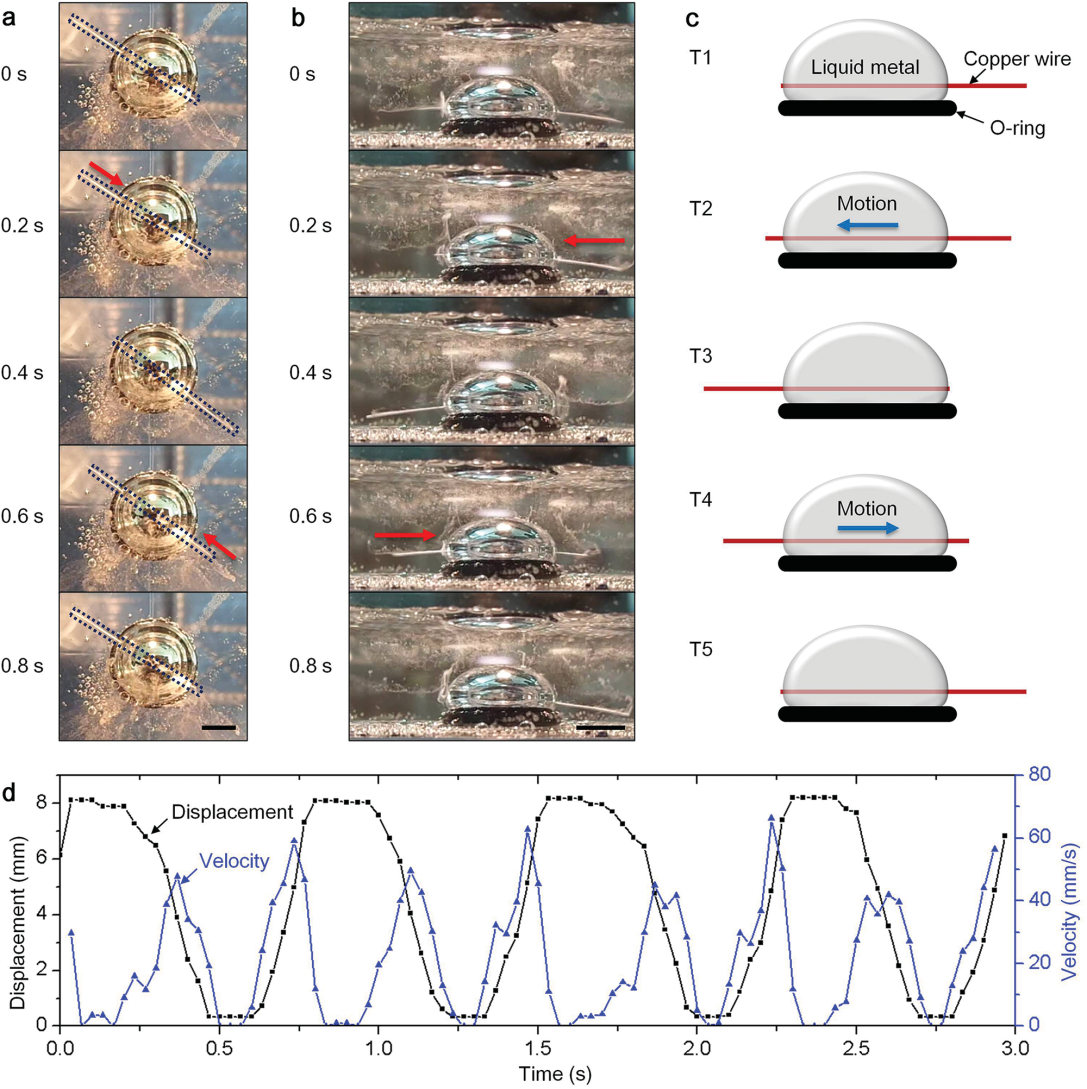
Oscillation behavior of copper wire in liquid metal machine. a) Top down view (scale bar, 5 mm; see Supporting Information Video S1). b) Side view (scale bar, 5 mm; see Supporting Information Video S2). c) Position of the copper wire during one period. d) Time evolution of the copper wire during 3 s.

To investigate the oscillation rhythm in detail, we collected the data for the time interval of the copper wire oscillation during 50 periods (**Figure** **2**). We defined four states of the copper wire during one period according to the time and position in order to facilitate the following discussion (Figure 2a). Statistics showed that the pausing time (processes A‐B and C‐D) varied with time. Occasionally, such pausing time could be up to minutes (Extended Data Figure S3 and Video S4, Supporting Information). The moving time (processes B‐C and D‐A) was almost the same (Figure 2b), which took about 0.22 s.

**Figure 2:**
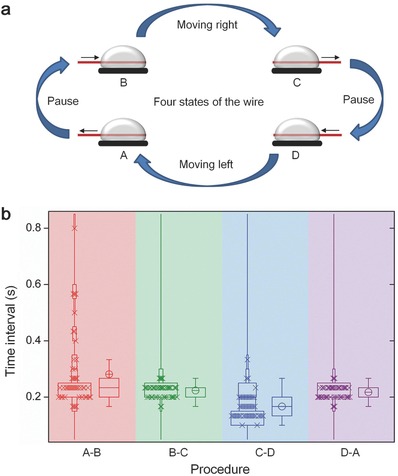
Statistic analysis of the oscillation phenomenon. a) Different states during a typical oscillation period. The copper wire moved to the left‐hand side (A), pausing for a while (B) before moving to the right‐hand side (C), pausing again (D) before moving left to state (A). b) Time interval between the four states during 50 period times. The box chart indicates the 25%, 50%, and 75% of the data (the crosses on the left represent the data). The whisker shows the outlier range with co‐efficient number to be 1.5. The circle indicates the mean of the data. The following box charts follow the same setting.

This oscillation behavior can be explained by the dynamic imbalance of the wetting force on two edges of the copper wire contacting liquid metal. The dragging force induced in three‐phase region competes on two edges and results in the oscillatory behavior (**Figure** **3**). The wetted copper wire surface was quite rough (Figure 3b) compared with bare copper wire (Figure 3a) since tiny solid Al granules would attach on it when moving through the liquid metal body. Assume that the copper wire was moving from left to right (Figure 3c), the left part would carry solution in the rough surface inside the liquid metal. On the other hand, the solution carried by the right part of the wire had reacted with Al granules, producing H_2_ in large quantities ($2\mathrm{Al}+2{\mathrm{OH}}^{-}+2H_{2}O\overset{}{\to }2{\mathrm{AlO}}_{2}^{-}+3H_{2}↑$). These H_2_ reduced the contact area between liquid and solid wire, reducing the dragging force along the wire. Thus, the dragging force on the left‐hand side becomes higher than that on the right‐hand side.

**Figure 3:**
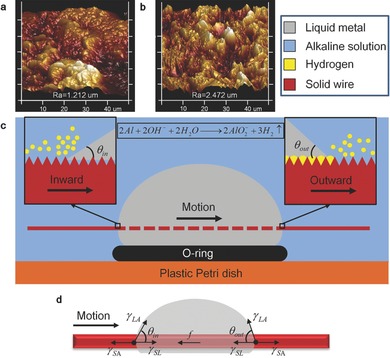
Interaction between copper wire, liquid metal, and alkaline solution. a) Surface structure of bare copper wire measured by atomic force microscope (AFM). The roughness is 1.212 μm. b) Surface structure of wetted copper wire measured by AFM. The roughness is 2.472 μm. c) Chemical reaction nearing contact lines. d) Force analysis of the copper wire.

The contact angle between copper wire and liquid metal machine can be described by the Cassie–Baxter equation,^15^, ^16^ i.e., $\cos\theta_{c}=f_{1}\cos\theta_{1}+f_{2}\cos\theta_{2}$, where *θ*_c_ represents the apparent contact angle and ƒ_1_ the total area of solid under the drop per unit projected area, with *θ*_1_ as the contact angle on a smooth surface of material 1. Likewise, ƒ_1_ is defined in a similar way.

Since the contact angle was determined just by the contact line region,^16^, ^17^ as depicted in Figure 3c, we could ignore other regions far from it. In our case, the contact angle at left part of contact line and right part are expressed as $\cos\theta_{\mathrm{in}}=f_{1}\cos\theta_{1}+f_{2}\cos\theta_{2}$ and $\cos\theta_{\mathrm{out}}=f_{1}^{*}\cos\theta_{1}+f_{3}\cos\theta_{3}$ respectively, with material 1 as solid wire, material 2 as alkaline solution, and material 3 as hydrogen. Since the generated hydrogen volume was 1000 times more than reacted alkaline solution (calculated from chemical reaction), the total area of solid contacting liquid metal per unit projected area on the right part ƒ_1_^*^ was smaller than left part ƒ_1_. Thus, $f_{1}\cos\theta_{1}$ is larger than $f_{1}^{*}\cos\theta_{1}$. Besides, it is obvious that ƒ_2_ was smaller than ƒ_1_. Since the surface tension of liquid metal was almost ten times higher than alkaline solution, we could assume *θ*_2_,*θ*_3_ to be π. Thus, $f_{2}\cos\theta_{2}$ is larger than $f_{3}\cos\theta_{3}$. As a result, $\cos\theta_{\mathrm{in}}$ is larger than $\cos\theta_{\mathrm{out}}$. Since the contact angle is the same along the contact line region between wetted copper wire and liquid metal,^17^ the dragging forces on the two edges can be expressed as follows^12^

(1) $$F_{\mathrm{in}}=\pi D_{\mathrm{in}}\left(\gamma_{\mathrm{LA}}\cos\theta_{\mathrm{in}}+\gamma_{\mathrm{SL}}-\gamma_{\mathrm{SA}}\right)$$

(2) $$F_{\mathrm{out}}=\pi D_{\mathrm{out}}\left(-\gamma_{\mathrm{LA}}\cos\theta_{\mathrm{out}}+\gamma_{\mathrm{SA}}-\gamma_{\mathrm{SL}}\right)$$

where *D* represents the diameter of solid wire.

We assume that the diameter on the left and right was almost the same, then the total driving force acting on the copper wire was expressed as

(3) $$F_{\mathrm{drive}}=\pi D\gamma_{\mathrm{LA}}\left(\cos\theta_{\mathrm{in}}-\cos\theta_{\mathrm{out}}\right)$$

For liquid metal without Al granules, the average adhesion force between liquid metal and wetted copper wire can be as high as 0.27 mN (Extended Data Figure S4, Supporting Information). The force enabled the wetted copper wire shooting through the liquid metal drop once it contacted the drop surface within 60 ms (Extended Data Figure S5 and Video S5, Supporting Information). The mass of wetted copper wire was 0.2 mg and thus according to Newton second law, i.e. *F = ma*, we got the initial transient acceleration as high as 1.35 m s^−2^. After the copper wire ran through the liquid metal drop, the wetting force balanced on two edges and the wire stopped gradually due to friction force, i.e., the viscous force between copper wire and surrounding liquids.

For liquid metal with Al granules, the imbalance of dragging force due to contact angle difference provides the driving force *F*_drive_. At the same time, the solution brought inside liquid metal would react with Al fiercely, producing H_2_. The H_2_ also functioned as a lubricating film between liquid metal and wire, reducing the friction force greatly.

For the pausing stage, it might be resulted from the delay of reaction. When the wire moved to one side, the solution brought inside liquid metal would take some time before reacting with Al granules. Before enough H_2_ was produced, the dragging force was unable to overpass the dragging force on the other side.

Apart from the above phenomenon, we found further interesting effect that by touching a steel needle on the liquid metal surface, the oscillation period can be shortened significantly (**Figure** **4** and Supporting Information Video S6). H_2_ would be generated on the steel needle fiercely due to the primary battery formed between steel and Al.^18^ Statistical analysis showed that the period shortened was mainly the pausing time (Figure 4b). This might be that the accelerated reaction speed reduced the time for the solution on the wire to react with Al. The moving time was shortened slightly as well. Copper wires with different length (15 mm, 25 mm) were also investigated for the same process (Extended Data Figure S6 and Videos S7 and S8, Supporting Information).

**Figure 4:**
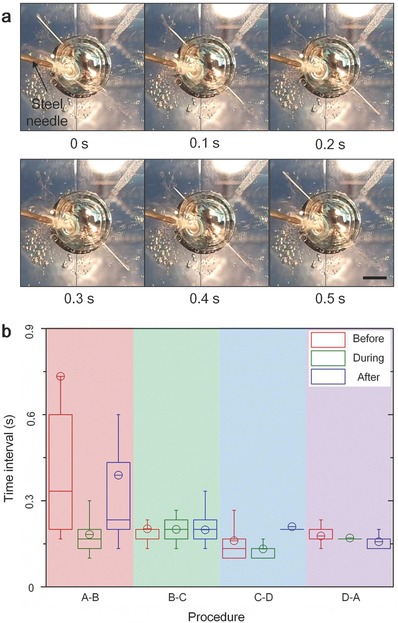
Oscillation period regulated by touching a steel needle on liquid metal surface. a) Top down view of oscillation with a steel needle contacting the liquid metal surface (scale bar, 5 mm; see Supporting Information Video S6). b) Comparison of time interval between the four states (Figure 2a) before, during, and after the needle contacting the liquid metal surface.

In summary, the present findings offer a unique strategy toward fabricating self‐fueled oscillator machines with no rigid bodies. The whole autonomous oscillation system was easily fabricated and reliable. The oscillation period can be regulated by simply contacting a steel needle to the surface of the liquid metal machine. These revealed fundamental phenomena and the mechanisms disclosed refresh the basic knowledge of classical oscillation and wetting effects and provide insight into further exploration. It also suggested an important platform for developing future complex hybrid machine structure made of soft and liquid mechanical systems together.

## Experimental Section

Fabrication of the liquid metal motor was similar with previous work.^13^ Liquid metal GaIn_10_ (10 wt% In) was injected onto a piece of Al foil in a 10 mL beaker filled with 0.5 m aqueous NaOH solution. The mass ratio of GaIn_10_ and Al foil was about 200:1. Liquid metal GaIn_10_ would gradually penetrate into Al grain boundaries.^19^ After 10 min, Al foil was totally decomposed into small granules by liquid metal. Some of the granules would float on liquid metal surface and accumulated together while others were dispersed inside liquid metal.^13^ The prepared liquid metal (≈0.4 mL) was then moved onto a rubber O‐ring (diameter 1 cm) in the case of self‐motion induced by surface tension gradient.^10^ The whole system was immersed inside 0.5 m aqueous NaOH solution in a square plastic Petri dish. Since the copper wire would be totally wetted by liquid metal after oscillation, we coated the copper wire (length 2 cm and diameter 0.19 mm) via GaIn_10_ in advance before inserting it inside the prepared liquid metal to shorten the fabrication time. Initially, the copper wire just stayed at one side and did not move. The wire was pulled out from the liquid metal and the inserting process was repeated several times until the surface was covered with enough tiny Al granules from the liquid metal. Oscillation then happened spontaneously with H_2_ bubbles rising from the copper wire surface constantly (Figure 1a,b).

## Supporting information

As a service to our authors and readers, this journal provides supporting information supplied by the authors. Such materials are peer reviewed and may be re‐organized for online delivery, but are not copy‐edited or typeset. Technical support issues arising from supporting information (other than missing files) should be addressed to the authors.

Supplementary

Supplementary

Supplementary

Supplementary

Supplementary

Supplementary

Supplementary

Supplementary

Supplementary
