# Supplementary material for: Liquid Metal Machine Triggered Violin‐Like Wire Oscillator
Source: Adv Sci (Weinh). 2016 Aug 17;3(10):1600212. doi: 10.1002/advs.201600212 (PMC5096059; doi:10.1002/advs.201600212)
Supplement: Supplementary file 1 — Supplementary [file ADVS-3-0b-s001.pdf]

Copyright WILEY-VCH Verlag GmbH & Co. KGaA, 69469 Weinheim, Germany, 2016.

ADVANCED SCIENCE
Open Access

Supporting Information

for *Adv. Sci.,* DOI: 10.1002/advs.201600212

Liquid Metal Machine Triggered Violin-Like Wire Oscillator

*Bin Yuan, Lei Wang, Xiaohu Yang, Yujie Ding, Sicong Tan, Liting Yi, Zhizhu He, and Jing Liu**

Supporting Information

Liquid Metal Machine Triggered Violin-like Wire Oscillator

Bin Yuan, Lei Wang, Xiaohu Yang, Yujie Ding, Sicong Tan, Liting Yi, Zhizhu He and *Jing Liu*[*]

Extended Data Figures and Legends:

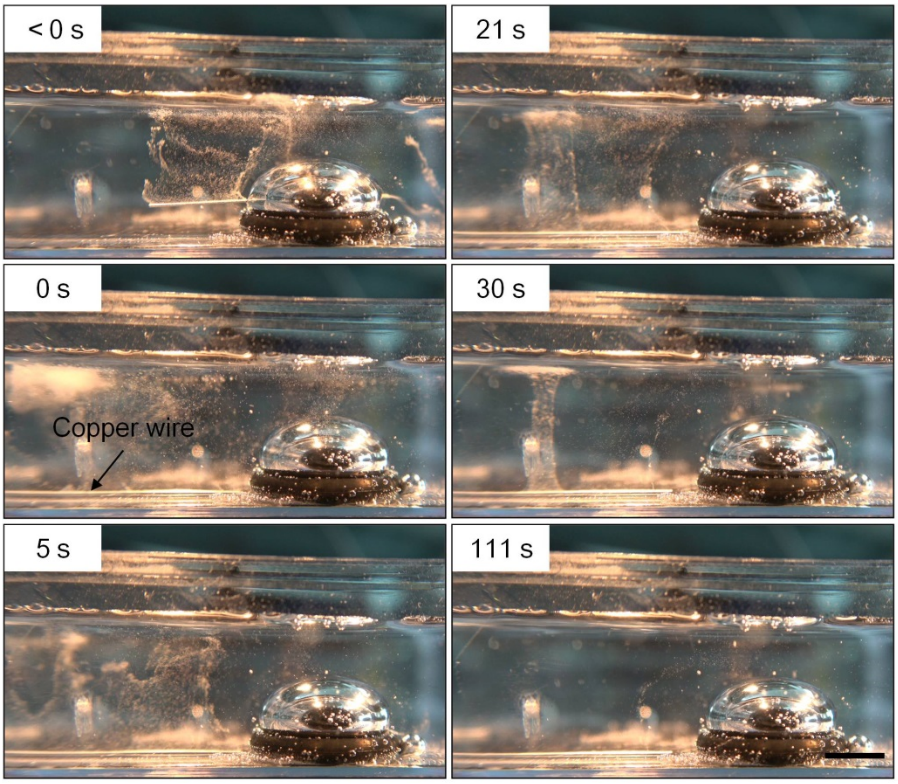

Extended Data Figure 1. H_2 generation on the copper wire after being pulled out of liquid metal (scale bar, 5mm; see Supplementary Video 3).

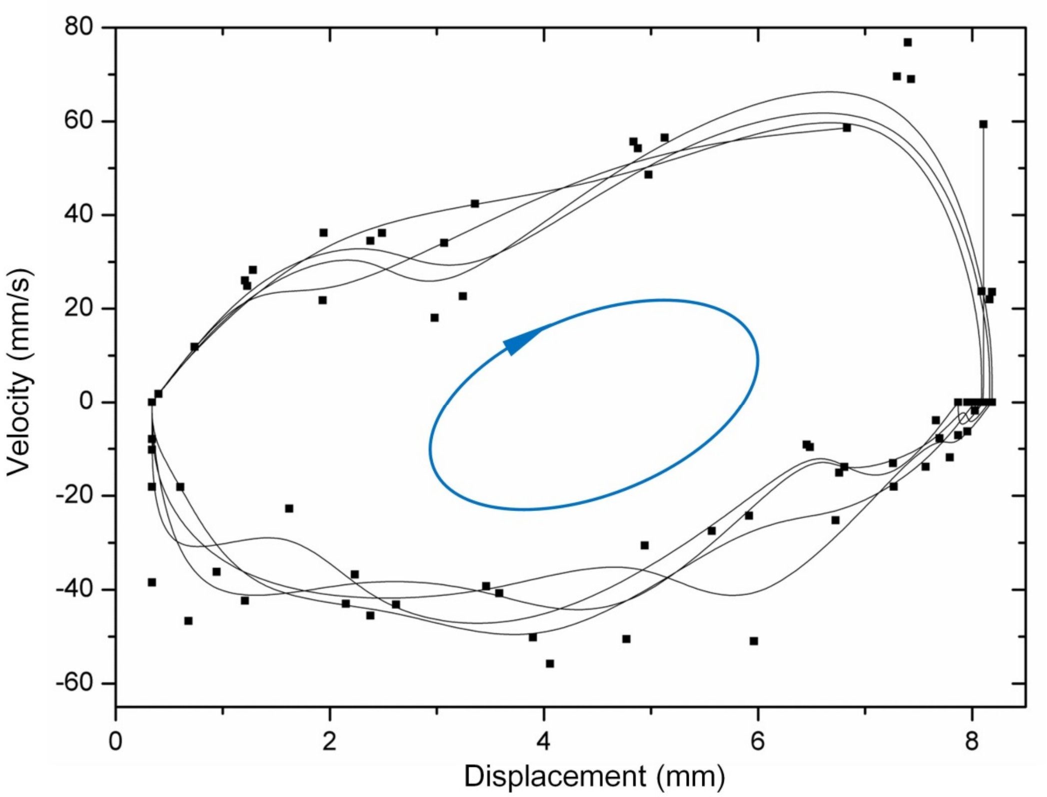

Extended Data Figure 2. Phase portrait of the oscillation process. The data were fitted by S-pline curve.

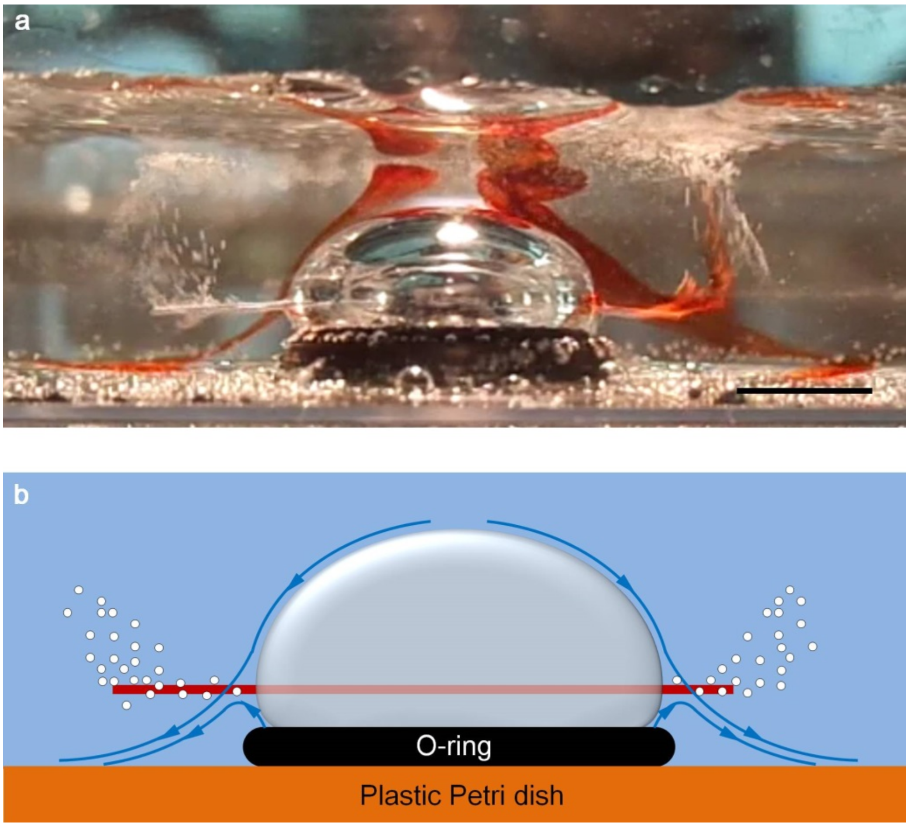

Extended Data Figure 3. Pausing state of the copper wire. a, Side view (scale bar, 5 mm; see Supplementary Video 4), red ink was dropped to reveal the flow field. b, The stream line on liquid metal surface.

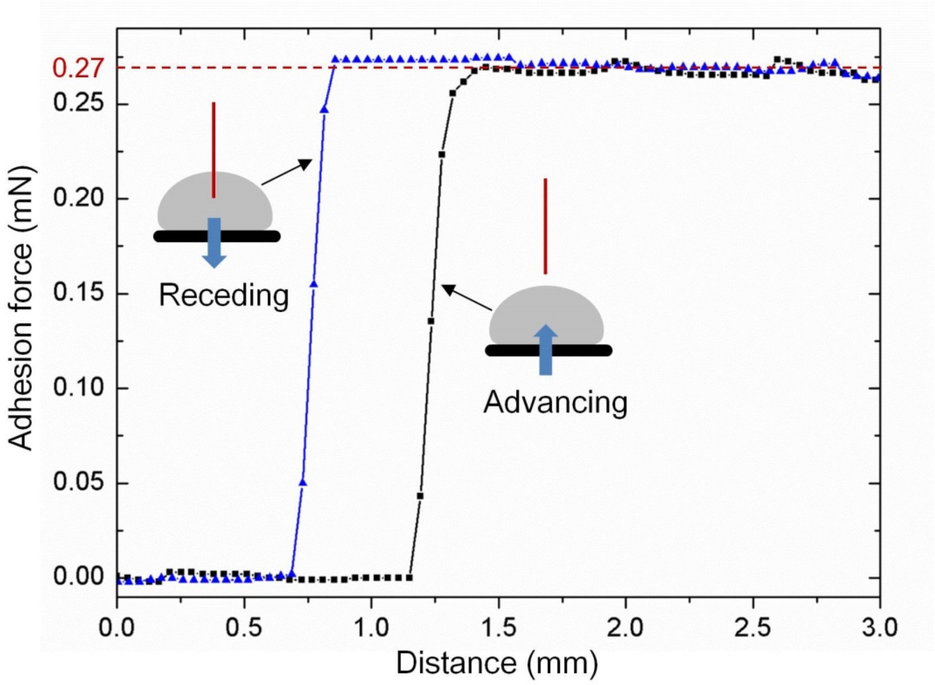

Extended Data Figure 4. Adhesion force between the wetted copper wire and liquid metal $GaIn_{10}$. The adhesion force was measured by Dynamic Contact Angle Meter and Tensionmeter.

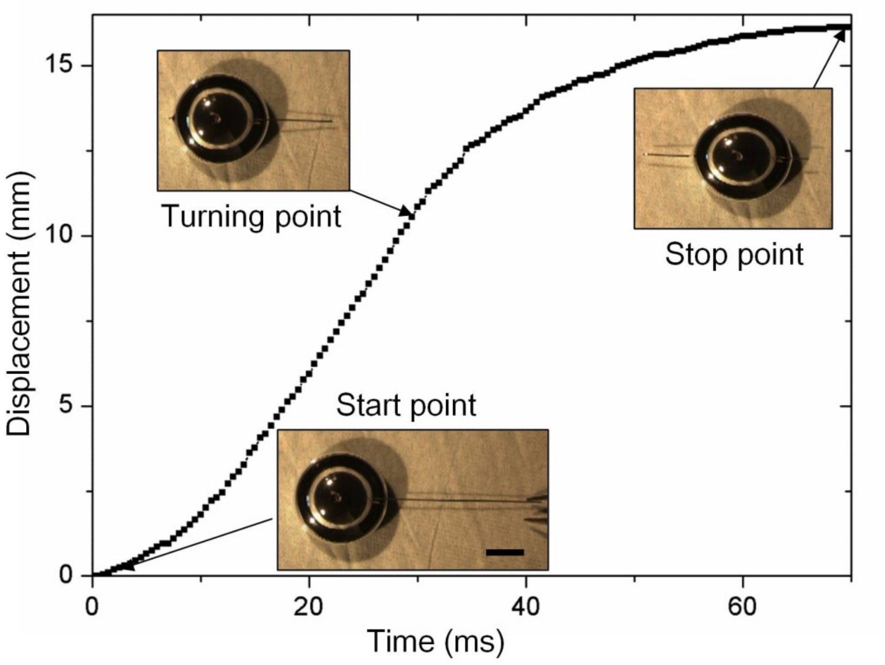

Extended Data Figure 5. Time evolution of a wetted copper wire swallowed by liquid metal $GaIn_{10}$. The start point refers to the wetted copper wire contacting the liquid metal, turning point as the copper wire runs out on the other side and stop point as the copper wire stops (scale bar, 5 mm; see Supplementary Video 5).

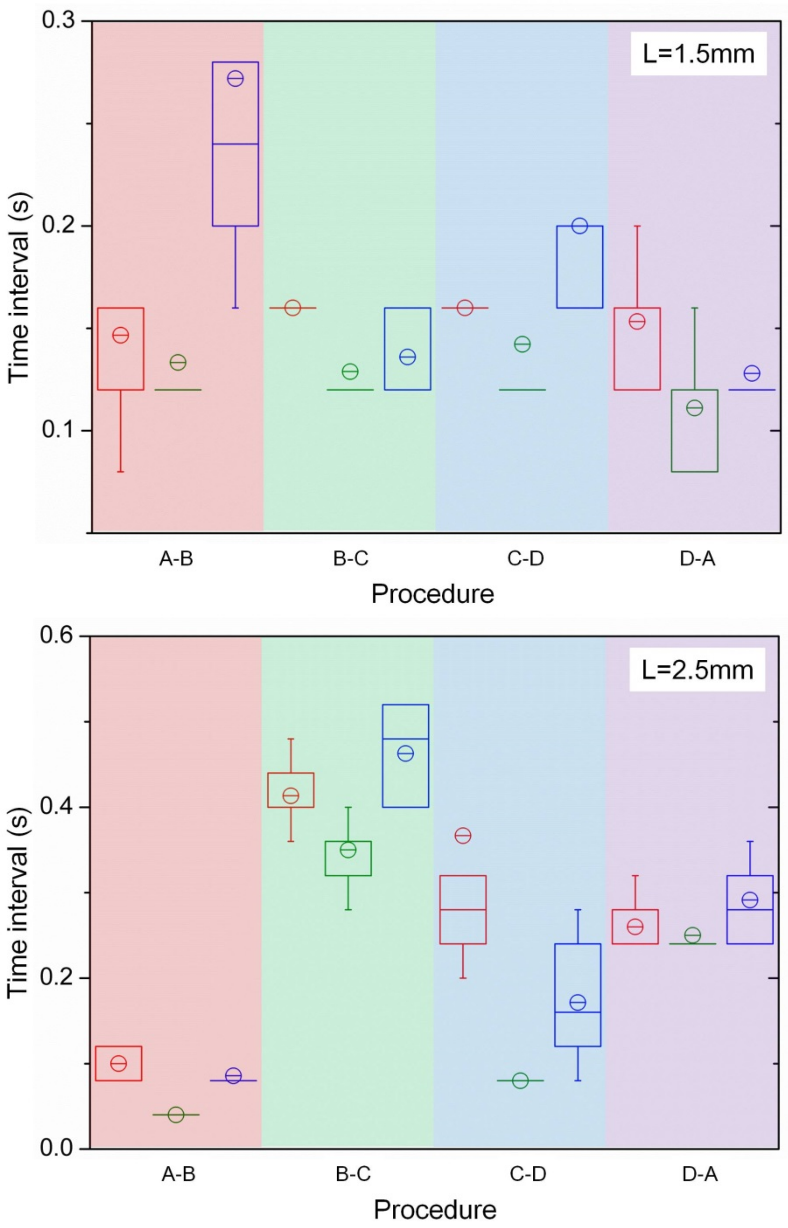

Extended Data Figure 6. Comparison of oscillation behavior before and after contacting steel needle for copper wire with different length (see Supplementary Video 7 and 8).

Supplementary Videos

Supplementary video 1. Top view of the self-powered oscillation phenomenon. The whole system was immersed in 0.5M aqueous NaOH solution filled in a square plastic Petri dish. The liquid metal machine (approximately 0.4 ml, mass ratio of GaIn10 and aluminum was about 200:1) was placed in a rubber O-ring (diameter 10.6 mm) on Petri dish floor. The copper wire (length 20mm, diameter 0.19 mm) was oscillating in liquid metal machine with hydrogen generated on wire surface. The video was taken from the top.

Supplementary video 2. Side view of the self-powered oscillation phenomenon. The whole system was immersed in 0.5M aqueous NaOH solution filled in a square plastic Petri dish. The liquid metal machine (approximately 0.4 ml, mass ratio of GaIn10 and aluminum was about 200:1) was placed in a rubber O-ring (diameter 10.6 mm) on Petri dish floor. The copper wire (length 20mm, diameter 0.19 mm) was oscillating in liquid metal machine with hydrogen generated on the wire surface. The video was taken from the side.

Supplementary video 3. Hydrogen generation on copper wire surface after pulled out of the liquid metal machine. The copper wire was pulled out of the liquid metal machine and placed on the Petri dish floor. Hydrogen was generating on copper wire surface. The middle part of copper wire generated hydrogen for a longer time. The video was taken from the side.

Supplementary video 4. Pausing state of the copper wire. The copper wire was pausing in the liquid metal machine. Red ink was dropped on the top and flows with solution. Hydrogen on copper wire surface was also taken aside by the flow. The video was taken from the side.

Supplementary video 5. Evolution of a wetted copper wire swallowed by liquid metal $GaIn_{10}$. A copper wire wetted by GaIn10 was released when contacting the liquid metal

surface. The copper wire ran through the liquid metal and stopped on the other side. High speed camera was used to capture the process from the top. The video was displayed 200 time slower than the actual speed.

Supplementary video 6. Oscillation period of a 20mm long copper wire regulated through touching a steel needle on liquid metal surface. The video was taken from the top.

Supplementary video 7. Oscillation period of a 15mm long copper wire regulated through touching a steel needle on liquid metal surface. The video was taken from the side.

Supplementary video 8. Oscillation period of a 25mm long copper wire regulated through touching a steel needle on liquid metal surface. The video was taken from the side.
